# Cloning and characterization of a glucosyltransferase from *Crocus sativus *stigmas involved in flavonoid glucosylation

**DOI:** 10.1186/1471-2229-9-109

**Published:** 2009-08-20

**Authors:** Ángela Rubio Moraga, Almudena Trapero Mozos, Oussama Ahrazem, Lourdes Gómez-Gómez

**Affiliations:** 1 Departamento de Ciencia y Tecnología Agroforestal y Genética, ETSIA, Universidad de Castilla-La Mancha, Campus Universitario s/n, Albacete, 02071, Spain; 2 Current address: Centro Regional de Investigaciones Biomedicas, C/Almansa 14, Albacete, 02006, Spain

## Abstract

**Background:**

Flavonol glucosides constitute the second group of secondary metabolites that accumulate in *Crocus sativus *stigmas. To date there are no reports of functionally characterized flavonoid glucosyltransferases in *C. sativus*, despite the importance of these compounds as antioxidant agents. Moreover, their bitter taste makes them excellent candidates for consideration as potential organoleptic agents of saffron spice, the dry stigmas of *C. sativus*.

**Results:**

Using degenerate primers designed to match the plant secondary product glucosyltransferase (PSPG) box we cloned a full length cDNA encoding CsGT45 from *C. sativus *stigmas. This protein showed homology with flavonoid glucosyltransferases. *In vitro *reactions showed that CsGT45 catalyses the transfer of glucose from UDP_glucose to kaempferol and quercetin. Kaempferol is the unique flavonol present in *C. sativus *stigmas and the levels of its glucosides changed during stigma development, and these changes, are correlated with the expression levels of CsGT45 during these developmental stages.

**Conclusion:**

Findings presented here suggest that CsGT45 is an active enzyme that plays a role in the formation of flavonoid glucosides in *C. sativus*.

## Background

Flavonols constitute a major class of plant natural products that accumulate in a wide range of conjugate structures. A large proportion of this diversity is due to the attachment of one or several sugar moieties at different positions. Besides providing beautiful pigmentation in flowers, fruits, seeds, and leaves [1], flavonoids also have key roles in signalling between plants and microbes, in male fertility of some species [2], in defence as antimicrobial agents and feeding deterrents [3], in UV protection [4], in the regulation of polar transport of auxins [5], and more recently, their role in cell cycle regulation in plants has been demonstrated [6,7]. There is increasing evidence to suggest that flavonoids, in particular those belonging to the class of flavonols (such as kaempferol and quercetin), are potentially health-protecting components in the human diet as a result of their high antioxidant capacity [8,9]. Therefore, flavonoids may offer protection against major diseases such as coronary heart diseases and cancer [10,11]. Flavonoids are present at relatively high concentrations in saffron, the dessicated stigma tissue of *C. sativus *[12,13]. Their antioxidant properties, along with their bitter taste, could qualify them as potential organoleptic agents of the spice [13-15]. In addition, they show anticonceptive and anti-inflammatory effects [16]. Nevertheless, the studies of these compounds in saffron stigma are scarce, and have only been analysed with some detail in tepals [17,18].

Flavonoid synthesis is organ- and tissue-dependent, and is affected by environmental conditions [19]. In the early steps of flavonoid biosynthesis, phenylalanine derived from the shikimic acid pathway is converted to coumaroyl-CoA by phenylalanine ammonia-lyase, cinnamate 4-hydroxylase, and 4-coumarate:CoA ligase. Chalcone synthase, the first committed enzyme for flavonoid biosynthesis, results in the condensation of coumaroyl-CoA with three molecules of malonyl-CoA from acetyl-CoA to form naringenin chalcone, which suffers further modifications that result in the synthesis of substitute flavones, flavonols, catechins, deoxyflavonoids, and anthocyanins. The flavonoid aglycones, which have a variety of glycosylation sites, are converted into glycon by glycosyltransferases.

In higher plants, secondary metabolites are often converted to their glycoconjugates, which are then accumulated and compartmentalized in vacuoles [20], while glycosylation of phytochemicals is known to alter their regulatory properties by causing enhanced water solubility and lower chemical reactivity. Glycosylation involves a UGT-catalysed transfer of a nucleotide diphosphate-activated sugar molecule to the acceptor aglycone [21]. The glycosylation reactions are catalysed by glycosyltransferases (GTases). Among these GTases, family 1 GTases (UGTs), commonly utilize small molecular weight compounds as acceptor molecule substrates and UDP-sugars as donors [22]. The first gene encoding a plant glycosyltransferase was isolated in *Zea mays*, during the analysis of the Bronze locus, which codes for an UDP-glucose:flavonol glucosyltransferase [23]. Since then, several clones have been characterized at a molecular level in a range of species including *Petunia hybrida *[24,25], *Vitis vinifera *[26], *Perilla frutescens *[27], *Allium cepa *[28], *Nicotiana tabacum *[29], *Arabidopsis thaliana *[30-34], *Dianthus caryophyllus *[35], *Beta vulgaris *[36], *Glycine max *[37]; *Pyrus communis *[38], *Oryza sativa *[39,40] and *Fragaria × ananassa *[41] among others.

Here the isolation of a UDP-glucose:flavonol glucosyltransferase from *C. sativus *stigmas using a degenerate PCR technique is reported. The substrate specificity analyses using recombinant protein indicated that *C. sativus *flavonol GT, CsGT45, was able to catalyse glucosylation of kaempferol and quercetin. Interestingly, *CsGT45 *was not expressed in *Crocus *species unable to accumulate kaempferol 7-*O*-glucosides in stigmas, suggesting the involvement of CsGT45 in the formation of kaempferol glucosides in the stigma tissue of *C. sativus*.

## Results

### Profile of flavonols accumulation during stigma tissue development

**Figure 1:**
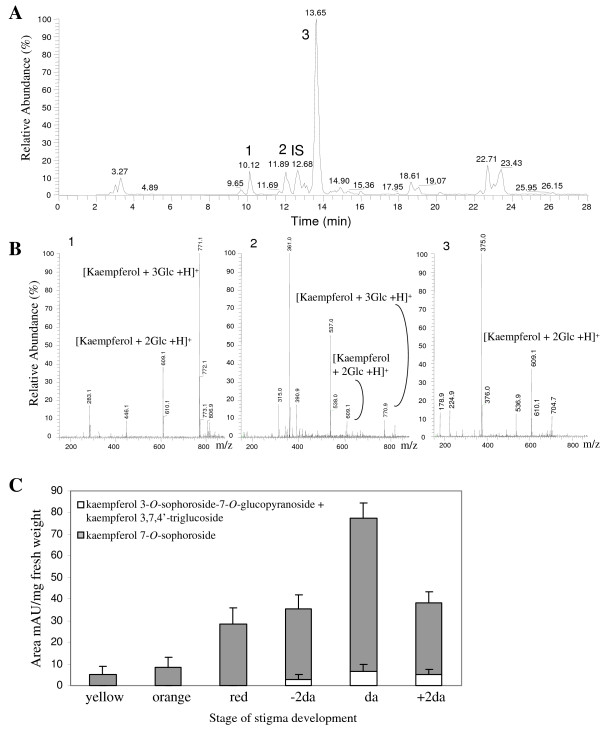
**Presence of flavonoid glucosides in *C. sativus *stigmas**. (A) HPLC-ESI-MS chromatogram of a MeOH extract of *C. sativus *stigmas at anthesis. Three flavonoid peaks, 1, 2, and 3 are denoted by arrows. The compound 4-methylumbelliferyl β-D-glucuronide was used as internal standard (IS). (B) Positive ion mass spectrum corresponding with the observed flavonoid peaks in A: 1, kaempferol 3-*O*-sophoroside-7-*O*-glucopyranoside; 2, kaempferol 3,7,4'-triglucoside, and 3, kaempferol 7-*O*-sophoroside acquired during the HPLC-ESI-MS analysis. (C) Relative kaempferol 3-*O*-sophoroside-7-*O*-glucopyranoside, kaempferol 3,7,4'-triglucoside and kaempferol 7-*O*-sophoroside levels at different stigma developmental stages.

In saffron, the flavonoids kaempferol 3-*O*-sophoroside-7-*O*-glucopyranoside and kaempferol 7-*O*-sophoroside were identified as abundant compounds [12,13], and more recently, a kaempferol tetrahexoside and kaempferol 3,7,4'-triglucoside have been tentatively identified as minor flavonoids in saffron [15], whereas quercetin and its glucosides have not been detected. Initially the content of flavonoids present in *C. sativus *stigma at anthesis was analysed by LC-ESI-MS (Figure 1A). In addition, six stigma developmental stages were selected and methanol extracts were analysed by HPLC. Under our experimental conditions, three significant flavonoids were evident in the HPLC chromatograms from extracts of *C. sativus *stigmas (Figure 1A). The retention times, the UV spectra and the LC-ESI-MS analysis on stigmas at anthesis allowed us to tentatively identify these flavonoids as 3-*O*-sophoroside-7-*O*-glucopyranoside, 3,7,4'-triglucoside and 7-*O*-sophoroside (Figure 1B). This compound was also characterized by NMR analysis and the obtained structural data correspond to those found in the literature [13]. The presence of all three flavonoids increased with stigma development and the increase for the two kaempferol triglucosides was equal. The relative levels of kaempferol 7-*O*-sophoroside, which reached the maximum levels at anthesis, were much higher than those observed for both kaempferol 3-*O*-sophoroside-7-*O*-glucopyranoside and kaempferol 3,7,4'-triglucoside, with relative high levels in the scarlet stages (-2da to +3da) (Figure 1C).

### Cloning and deduced structure of CsGT45

To identify flavonoid glucosyltransferases from *C. sativus *stigmas, a homology-based strategy was used, taking advantage of specific glycosyltranferase motifs located in the C-terminus region [42]. A cDNA population was prepared by reverse transcription of poly (A)^+ ^from total RNA isolated from *C. sativus *stigmas at anthesis, which showed the highest levels of kaempferol glucosides. DNA fragments were amplified by degenerate primers and the obtained products were cloned and analysed. Sequencing of one PCR product revealed homology to glycosyltransferases. The sequence information from this clone, *CsGT45*, allowed the design of PCR specific primers to obtain the full-length transcripts. We performed 5' and 3' RACE using poly(A)^+ ^from *C. sativus *stigma as a template. The GTase gene obtained (1674 bp, Gen Bank FJ194947) was intronless, containing a putative open reading frame of 1500 bp encoding 500 amino acid residues with a calculated molecular mass of 55.42 kDa and a pI of 5.19.

Because *C. sativus *is a triploid, we employed *in silico *screening of a large stigma cDNA EST database [43] as an effective method for identification of potential *CsGT45 *alleles. We identified three EST clones with 98% identity in 611 bp (EX147039.1), 98% identity in 264 bp (EX144545.1) and 84% identity in 426 bp (EX148389.1). The first two ESTs correspond to *CsGT45*, and the third could correspond to a *CsGT45 *allele.

The carboxyl terminal of the protein contained the plant secondary product glycosyltransferase (PSPG) box signature motif. Analysis of CsGT45 sequence for N-terminal targeting signal or C- terminal membrane anchor signal using SignalP and TMpred web-based programmes predicted CsGT45 to be non-secretory with an absence of predicted signal peptides or transmembrane signals [44].

**Figure 2:**
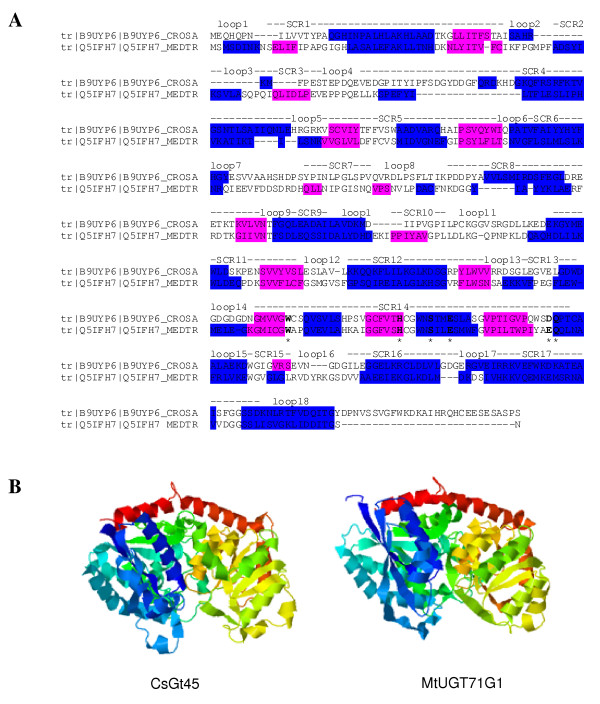
**Amino acids sequence alignment of CsGT45 against MtUGT71G1 and structures comparison**. (A) The alignment was performed guided by conservation of secondary structure, predicted for CsGT45 (B9UYP6) and observed from the solved crystal structure of MtUGT71G1 (Q5IFH7). α-helices are highlighted in blue and β-strands in pink. Structurally conserved regions (SCRs) are highlighted by dots above the alignment. Loops are numbered and named above the alignment. The amino acids residues within the PSPG motif that interact in MtUGT71G1 with the sugar donor are marked with starts. (B) Ribbon diagrams showing the conserved secondary and tertiary structure of MtUGT71G1 (right) used as template for modelling of CsGT45 and the constructed model (left).

For comparative modelling, CsGT45 was aligned with MtUGT71G1, whose crystal structure has recently been solved [45]. CsGT45 displayed 18% overall identity with MtUGT71G1 (Figure 2A). A molecular model of CsGT45 was constructed from the structural alignment. Structurally conserved regions of the CsGT45 model were built from the crystal structure of MtUGT71G1 using the Pyre server [46] (Figure 2B). In plant GTs, the most common sugar donor is UDP-Glc. Several conserved residues, most of which are found in the PSPG motif of plant UGTs, interact with the sugar donor [22]. The conserved residues involved in the interaction with UDP-Glucose in MtUGT71G1 are also conserved in CsGT45, with the exception of the E381 residue that in CsGT45 is aspartate residue D385, which is also found in the characterized VvGT1 [22].

**Figure 3:**
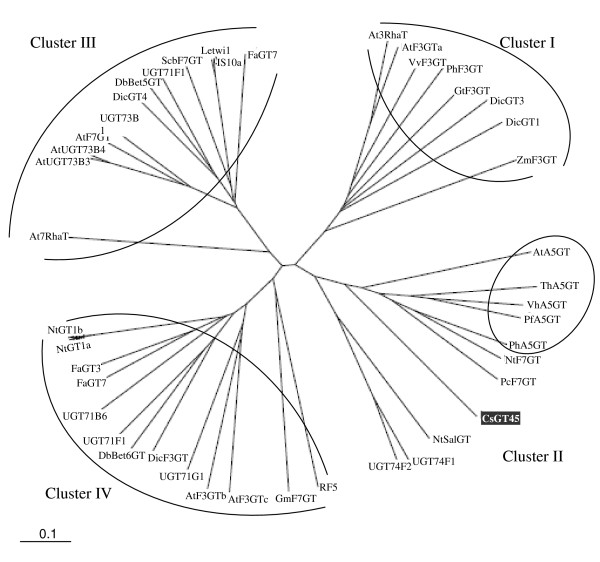
**Unrooted phylogenetic tree of the GTases based on amino acid sequence similarity**. GenBank accession numbers and sources for the respective protein sequences are: CsGT45 (FJ194947) from *Crocus sativus*; flavonoid 3-*O*-glucosyltransferases from *Arabidopsis thaliana *(AAD17392), AtUGT73B4 and (At5G17050), At GT; *Zea mays *(X13502), ZmF3GT; from *Vitis vinifera *(AAB81682), VvF3GT; from *Fragaria × ananassa *(BAA12737), GtF3GT; from *Dianthus caryophyllus *(BAD52005), DicGT3 and (BAD52003), DicGT1; At3RhaT, flavonol 3-O-rhamnosyltransferase from *Arabidopsis thaliana *(At1g30530); At7RhaT, flavonol 7-O-rhamnosyltransferase from *Arabidopsis thaliana *(NP_563756); flavonoid 7-*O*-glucosyltransferases from *Scutellaria baicalensis *(BAA83484), ScbF7GT; *Pyrus communis *(AAY27090), PcF7GT; from *Nicotiana tabacum *(BAB88935), NtF7GT from *Arabidopsis thaliana *(AAR01231), AtF7GT; NtSalGT, salicylic acid glucosyltransferase from *Nicotiana tabacum *(AAF61647); AtUGT73B3, pathogen-responsive glucosyltransferase from *Arabidopsis thaliana *(AAD17393); DicGT4, chalcononaringenin 2'-*O*-glucosyltransferase (BAD52006) from *Dianthus caryophyllus*; DbBet5GT, betanidin-5-*O*-glucosyltransferase from *Dorotheanthus bellidiformis *(CAB56231); UGT74F1, UGT74F2, and UGT73B1, flavonoid glucosyltransferases from *Arabidopsis thaliana *(AAB64022.1), (AAB64024.1) and (At4g34138); Letwi1, wound-inducible glucosyltransferase from *Solanum lycopersicum *(CAA59450); NtIS5a, immediate-early salicylate-induced glucosyltransferase from *Nicotiana tabacum *(AAB36653); FaGT7, multi-substrate flavonol-*O*-glucosyltransferase (ABB92749); AtF3GTb, putative flavonol 3-*O*-glucosyltransferases from *Arabidopsis thaliana *(NP_180535.1), AtF3GTc and (NP_180534.1), from *Petunia hybrida *(AAD55985), PhF3GT; from *Gentiana triflora *(BAA12737), GtF3GT; from *Dianthus caryophyllus *(BAD52004), DicF3GT; DbBET6GT, betanidin 6-*O*-glucosyltransferase from *Dorotheanthus bellidiformis *(AAL57240); UGT71B6, glucosyltransferase from *Arabidopsis thaliana *(AB025634); FaGT3 and FaGT7, flavonol-*O*-glucosyltransferases from *Fragaria × ananassa *(AAU09444) and (ABB92748); NtGT1a and NtGT1b, broad substrate specificity glucosyltransferases from *Nicotiana tabacum *(BAB60720) and (BAB60721); AtA5GT, glucosyltransferase from *Arabidopsis thaliana *(AAM91686); anthocyanin 5-*O*-glucosyltransferases from *Torenia hybrida *(BAC54093), ThA5GT; from *Verbena hybrida *(BAA36423), VhA5GT; from *Perilla frutescens *(BAA36421), PfA5GT; from *Petunia hybrida *(BAA89009), PhA5Gt; UGT71F1, regioselective 3,7 flavonoid glucosyltransferase from *Beta vulgaris *(AY526081); UGT73A4, regioselective 4',7 flavonoid glucosyltransferases from *Beta vulgaris *(AY526080); UGT71G1, triterpene glucosyltransferase from *Medicago truncatula *(AAW56092). The horizontal scale shows the number of differences per 100 residues derived from the ClustalW alignment.

Comparison of the predicted amino acid sequence with that of other glycosyltransferases reveals overall positional identities of 44% with *Pyrus communis *flavonoid 7-*O*-glucosyltransferase (AAY27090.1), 41% with *Arabidospis *flavonoid 3-*O*-glucosyltransferase (At5g17050) and flavonoid 7-*O*-glucosyltransferase NtF7GT (*Nicotiana tabacum*, BAB88935). The phylogenetic tree based on deduced amino acid sequences if plant GTases is shown in Figure 3. Currently, GTases function and specificity cannot be fully predicted based on sequence information alone. However, the phylogenetic tree of functionally characterized GTases showed several clusters, which could be characterized by the specificity of the flavonoid glycosyltransferase activities of enzymes involved therein. Cluster I is characterized by flavonoid 3-*O*-glycosyltransferases, cluster III mainly contains flavonoid 7-*O*-glycosyltransferases, and cluster IV contains broad substrate GTases. Cs45GT is included in cluster II, which contains anthocyanin 5-*O*-glucosyltransferases (A5GT), like VhA5GT, PfA5GT and PhA5GT which activities have been tested *in vitro *[25,27] and other GTases with a broad substrate specificity that are not involved in the biosynthesis of anthocyanins, like UGT74F1 and UGT74F2 from *Arabidopsis*, which produced distinct multiple glucosides of quercetin *in vitro *[47], while *in vivo *act as anthranilate glycosyltransferases [48] and GTases implicated in salicylic acid metabolism, like NtSalGT that reacts on several phenolic compounds *in vitro *[49]. NtF7GT from *Nicotiana *that reacts on the 7-hydroxyl group of flavonol and 3-hydroxyl group of coumarin [29] and PcF7GT from *Pyrus communis *that reacts on the 7-hydroxyl group of flavonol [38]. Therefore, CsGT45 was presumed to encode a flavonoid GTase in *C. sativus *stigmas and was subjected to further analyses.

### Biochemical characterization

**Figure 4:**
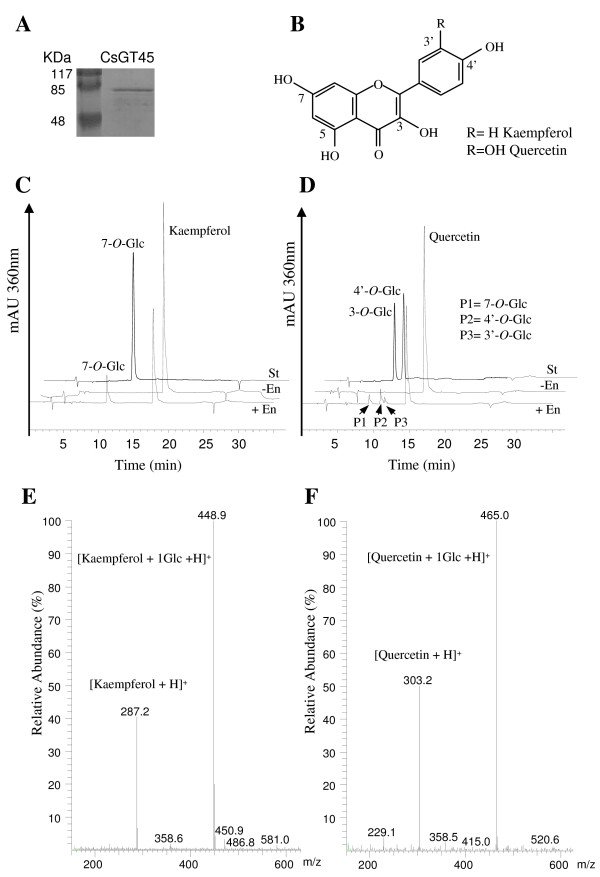
**The glutathione S-transferase-CsGT45 fusion protein shows activity toward flavonoids**. (A) The recombinant CsGT45 was analyzed using 10% (w/v) SDS-PAGE, and visualized with Coomassie staining. (B) Chemical structures of the flavonoids kaempferol and quercetin. (C) HPLC analysis of CsGT45 activity toward kaempferol. (D) HPLC analysis of CsGT45 activity toward quercetin. The obtained products, P1, P2, and P3 are denoted by arrows. (E) Positive ion mass spectrum of kaempferol 7-*O*-glucoside acquired during the HPLC-ESI-MS analysis. (F) Representative positive ion mass spectrum obtained for quercetin 7-*O*-glucoside, quercetin 4'-*O*-glucoside and 3'-*O*-glucoside acquired during the HPLC-ESI-MS analysis of each reaction product. Abbreviations: St, flavonol standard; -E, minus enzyme; and +E plus enzyme.

To identify the function of CsGT45, the full-length open reading frame was cloned into the expression vector pGEX-5T-3 for heterologous protein expression in *E. coli*. The recombinant protein was affinity purified on a glutathion sepharose column that binds the protein's N-terminal GST-tag (Figure 4A). Due to its homology with other flavonoid glycosyltransferases, CsGT45 was expected to glucosylate flavonoids. Activity tests were performed with UDP-Glucose and the flavonols quercetin and kaempferol (Figure 4B). CsGT45 forms monoglucosides on the 7- hydroxyl group of kaempferol (Figure 4C and 4E), whereas over quercetin forms monoglucosides on the 7-, 3'-, and 4'-hydroxyl groups of quercetin (Figure 4D and 4F). Glucosylation positions of the kaempferol and quercetin reaction products were assigned based on the hypsochromic shift data [50], comparison with published data [31,47,51] and when available, using authentic reference compounds. Flavonols have two absorption maxima: Band I (350–380) and Band II (240–280) corresponding to the B- and A-ring, respectively. Conjugation of 3-, 5-, or 4'-hydroxyl groups causes a Band I hypsochromic shift, which is larger for a 3-substitution (12–17 nm) than a 4'-conjugation (3–5 nm). The maximum absorbances of kaempferol were 266 and 368, and those of the kaempferol reaction product were 268 and 368 nm. The lack of a hypsochromic shift between substrate and reaction product strongly suggests that glycosylation occurred at the hydroxyl group of C-7, which was confirmed by comparison with an authentic reference standard (Figure 4C). For quercetin (256, 372) only the product P1 (256, 372) did not show a hypsochromic shift (Figure 4D) suggesting conjugation at the 7-hydroxyl group. P2 (254, 368) showed a Band I hypsochromic shift of 4 nm suggesting conjugation at the 4'-hydroxyl group, which was confirmed by comparison with an authentic reference standard (Figure 4D). The product P3 (252, 370) was tentatively assigned to quercetin 3'-*O*-glucoside based on comparison of related flavonoid product elution profiles [31,47], and by the lack of coincidence with the quercetin 3-*O*-glucoside standard regarding spectral data and elution time (Fig 4D). When longer incubation times (60 min) and higher substrate concentration (100 mM) of kaempferol or quercetin were used the formation of one diglucoside was observed for each flavonoid (data not shown). Other compounds, i.e. trans-cinnamic acid, sinapic acid, crocin, IAA and abscisic acid were assayed, but no activity was detected with any of these substrates. The results obtained suggest that CsGT45 acts on flavonols *in vivo*. The kinetic parameters for the individual glucosides formed were determined at variable concentrations of quercetin and kaempferol. The *K*_cat _and *K*_m _values are described in Table 1. The *V*_max_/*K*_m _ratios clearly demonstrate that CsGT45 exhibits the highest specificity towards 7-OH of kaempferol (100%), followed by the 7-OH and 4'-OH of quercetin (20.5 and 9.1%, respectively), and low affinity toward the 3'-OH (3.1%).

**Table 1:** The kinetic parameters *K*_m _and V_max _and the relation (V_max_/*K*_m_) of CsGT45, toward kaempferol and quercetin with a fixed UDPG concentration.

**substrate**	***K*_m _(μM)**	**V_max _(pkat/mg protein)**	**V_max_/*K*_m_**
Kaempferol 7-OH	15.6 ± 1.2	366 ± 19.8	23.46
Quercetin 4'-OH	86.95 ± 8.4	186 ± 12.4	2.14
Quercetin 3'-OH	30.3 ± 3.6	22.9 ± 2.6	0.75
Quercetin 7-OH	21.50 ± 2.3	104 ± 5.46	4.83

Enzyme assays were carried out using purified CsGT45 (7 μg), substrate (20 to 100 μM) and UDP-glucose (2.5 mM). Reactions mixtures were incubated at 30°C, and performed in triplicate.

The kinetic constants for UDP-glucose were also calculated. Different concentrations of UDP-glucose were assayed keeping the level of kaempferol constant. UDP-glucose showed a *K*_m _of 0.6 mM and a *V*_max _of 2.9 nkat/mg, thus suggesting that glucose is a good substrate for CsGT45.

### Spatial and developmental expression

**Figure 5:**
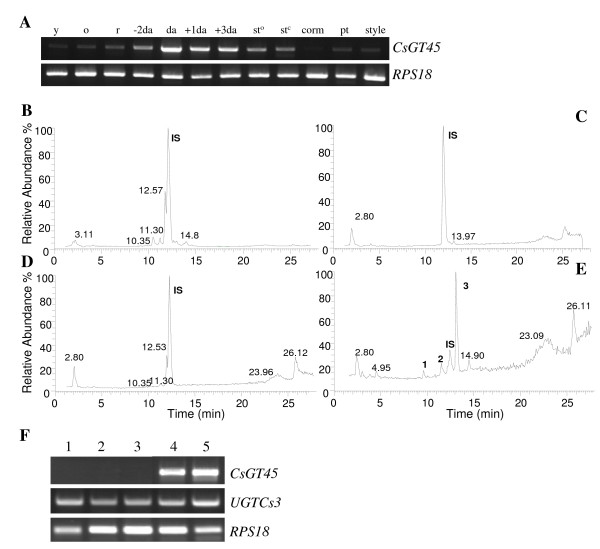
**Expression analysis of *CsGT45 *in plant tissues**. (A) The level of *CsGT45 *was analysed in the stigma tissue of *C. sativus *in different developmental stages: yellow (y), orange (o), red (r), two days before anthesis (-2da), anthesis (da), one day after anthesis (+1da), and three days after anthesis (+3da), and in closed and open stamen (st^c ^and st^o^), corm, tepals (pt) and style. Equal amounts of total RNA were used in each reaction. The levels of the constitutively expressed RPS18 coding gene were assayed as controls. (B) HPLC-ESI-MS chromatograms of MeOH extract of *C. cancellatus *stigmas at anthesis. (C) HPLC-ESI-MS chromatograms of MeOH extract of *C. niveus *at anthesis. (D) HPLC-ESI-MS chromatograms of MeOH extract of *C. speciosus *stigmas at anthesis. (E) HPLC-ESI-MS chromatograms of MeOH extract of *C. cartwrightianus *stigmas at anthesis. The peaks 1, kaempferol 3-*O*-sophoroside-7-*O*-glucopyranoside; 2, kaempferol 3,7,4'-triglucoside; and 3, kaempferol 7-*O*-sophorosid. The compound 4-methylumbelliferyl β-D-glucuronide was used as internal standard (IS). (F) Transcript levels of *CsGT45 *in the stigma tissue of different *Crocus *species: 1, *C.niveus*; 2, *C. cancellatus*; 3, *C. speciosus*; 4, *C. sativus *and 5, *C*. *cartwrightianus*. To ensure the detection of the transcripts, 40 PCR cycles were carried out.

The spatial and temporal expression pattern of *CsGT45 *was studied by RT-PCR throughout stigma development. Analyses were performed with RNA isolated from different stages of stigma development, i.e. flowers containing yellow, orange and red stigmas, which are characterized by the presence of immature anthers, and small tepals that do not show the characteristic purple coloration of *C. sativus*. These immature flowers are contained inside perianth tubes that elongate as flowers develop inside. Only when flowers are completely developed do they emerge from the perianth tubes and open when anthesis (da) occurs a few days later. Upon emerging, all flowers exhibited purple tepals and scarlet stigmas (-2da to +3da). The RT-PCR analysis revealed that *CsGT45 *expression is developmentally regulated. The *CsGT45 *transcript level in the yellow and orange stages was low, but increased from the red stage, and reached a peak at anthesis (Figure 5A). The expression of the *CsGT45 *was also examined in different tissues. The expression in flower tissues showed that *CsGT45 *transcripts were present in pollen, tepals and styles at low levels whereas expression in corms was practically undetectable under these conditions (Figure 5A).

The high expression levels of *CsGT45 *transcripts in the stigma tissue and its *in vitro *activity suggested that CsGT45 was associated with the observed kaempferol glucosylation in the stigma tissue. To investigate further such correlation the expression levels of *CsGT45 *were investigated in the stigma tissue of *Crocus *species in which kaempferol with substitutions in the 7-OH position were not detected (Figure 5B–D). These three *Crocus *species showed reduced flavonoid levels in comparison with *C. sativus*. In *C. niveus *we were unable could to detect kaempferol glucosides, in *C. speciosus *and *C. cancellatus *(Figure 5B and 5D) a kaempferol treahexoside was identified at position 10.35. This compound, substituted at position 3, has been also identified in *C. sativus *as a minor flavonoid [15]. The expression of *CsGT45 *was not detected in the stigma tissue of *C. niveus*, *C. speciosus *and *C. cancellatus *(Figure 5F), while was present in the stigma tissue of *C. sativus *and *C*. *cartwrightianus *that accumulate kaempferol with substitutions in the 7-OH position (Figure 1A and Figure 4E). By contrast, the expression of *UGTCs3*, a GTase previously identified in *C. sativus *stigmas [52] was detected in all the species (Figure 5F). The absence of *CsGT45 *expression in the stigma tissue of of *C. niveus*, *C. speciosus *and *C. cancellatus *suggests a role of CsGT45 in the accumulation of specific kaempferol glucosides in the stigma of *Crocus *species.

### Unaltered Expression of CsGT45 under stress conditions

**Figure 6:**
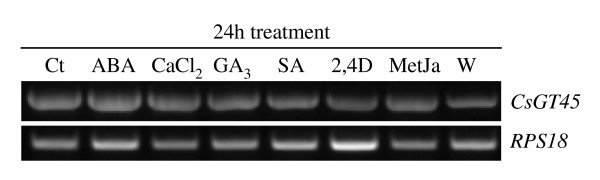
**Transcript levels of *CsGT45 *in response to different treatments**. Untreated control leaves (Ct), treated leaves with abscisic acid (ABA), calcium chloride (CaCl_2_), giberellic acid (GA_3_), potassium salicylate (SA), 2,4-dichlorofenoxiacetic acid (2,4D), methyl jasmonate (MetJa) and wounded leaves of *C. sativus *were collected 24 hr after treatment and total RNA extracted for *CsGT45 *expression analysis.

Several studies have shown that GTases are induced by a variety of stresses, including: salicylic acid [49,53], auxin [54], methyl jasmonate [55] and wounding [56]. To determine whether the gene expression levels of *CsGT45 *were influenced by exogenous hormones or by other stimuli such as drought stress and wounding, total RNA was isolated from treated leaves and used as template in the RT-PCR reactions. The expression of the gene was not altered 24 hours after the treatments (Figure 6). Shorter times were also tested with the same results (data not shown). Exogenous JA, ABA, GA_3_, or 2,4D did not significantly promote the expression of the genes (Figure 6). Drought, wounding and SA failed to affect the expression levels of *CsGT45 *(Figure 6).

## Discussion

In general, GTases that use secondary metabolites as substrates are minor constituents in plant cells [21]. Although many of these enzymes have been isolated from several plant species and assayed *in vitro*, in many cases their roles in the secondary metabolism of these plants are still unknown.

The saffron CsGT45 protein belongs to glucosyltransferase family 1, as do most of the UGTs involved in plant secondary metabolism. This protein possessed a PSPG box with a conserved sequence of 45 amino acid residues and showed specificity towards flavonoid aglycones. This protein has no signal sequence, nor any clear membrane-spanning or targeting signals, as the plant glycosyltransferases identified to date [57]. This suggests that these enzymes function in the cytosol, although within that compartment the proteins may associate as peripheral components of the endomembrane system, as previously suggested [58]. Sequence analysis showed CsGT45 as being most closely related to the *Pyrus communis *flavonoid 7-*O*-glucosyltransferase and belonging to the same clade of the phylogenetic tree, in which other glucosyltransferases of flavonoids attach sugars without high regiospecificity. The presence in this clade of A5GT enzymes suggest a common ancestral gene for all these GTases, where the A5GTs enzymes showed a strict substrate specificity [25,27,59], and seem to have evolved to a more specific function.

Plant secondary product glycosyltransferases have been reported to exhibit a rather strict regioselectivity towards the position of the sugar attachment [21]. The most common site on the flavonol molecule for glycosyl addition is carbon 3 of the C-ring, although other sites, especially the hydroxyl at carbon 7, are often substitutes [60]. However, in proportion, there are few studies on the enzyme activity and genes implicated in the catalysis of the 7-*O*-glucoside reaction. Many plant GTases recognize quercetin as an acceptor when assayed *in vitro*, and some others can glucosylate multiple hydroxyl groups of the aglycone and even form diglucosides in some cases [28-31,36,41,56]. In *Arabidopsis*, from ninety one GTases analyzed for their activity toward quercetin, 29 enzymes showed catalytic activity, and four recognize three sites [31]. Analysing the activity of some enzymes related to CsGT45, the *Arabidopsis *enzyme UGT74F1, glycosylated the 3'-OH, 4'-OH and 7-OH positions of quercetin [47]. We have observed similar activity for CsGT45 toward quercetin, but with a preference for the 7-OH position (*K*_m _21.5 μM). However, CsGT45 showed high regioselectivity toward kaempferol, and the same was reported for NtGT7 [29], present in the CsGT45 cluster. This feature is characteristic for several 7-*O*-glucosyltransferases present in cluster III and IV and distantly related to CsGT45 [28,37,39,40,56]. The *K*_m _value of kaempferol with CsGT45 is 15.6 μM, indicating its sufficient affinity to the substrate. These *K*_m _values were the same as other plant GTases reported [29,56], suggesting that these substrates are reasonable acceptors for CsGT45. Moreover, the CsGT45 enzyme did not react on other OHs of kaempferol, indicating that in this case the regiospecificity of the glucosylation is strictly determined. It has recently been reported that the hydroxylation pattern in ring B of the acceptor molecule can influence product specificity. UGT73A4 from *Beta vulgaris *accepts the positions 4' and 7 of flavonols. If a hydroxy group is present at position 3' (e.g. quercetin), 4'-*O*-glucosides are preferentially formed. If the hydroxy group is missing (e.g. kaempferol), the enzyme produced 7-*O*-glucosides [36]. Perhaps the differences observed for CsGT45 towards quercetin or kaempferol are due to this fact.

Because quercetin and kaempferol are substrates for CsGT45 *in vitro*, it is reasonable to propose that this enzyme is involved in the glucosylation of phenolics in the stigma tissue. Analysis of the flavonoid fraction from saffron revealed the unique presence of kaempferol [15]. The gene expression pattern of *CsGT45 *correlates with high levels of kaempferol glucosides in the stigma tissue. Interestingly the three main flavonoid glycosides detected in the stigma at anthesis were kaempferol 3-*O*-sophoroside-7-*O*-glucopyranoside, kaempferol 3,7,4'-triglucoside and kaempferol 7-*O*-sophoroside. CsGT45 was found to be active on the C-7 position of kaempferol, with the production of a monoglucoside and probably a diglucoside under the experimental conditions tested. Since kaempferol-7-*O*-sophoroside was the main flavonoid in the stigma tissue, we can speculate whether CsGT45 uses UDP-sophoroside as a sugar donor. However, UDP-Glc does appear to be a good donor and the sugar donor preference of a specific GT is often very narrow, showing little or no activity with alternative sugars [22]. By using a molecular modelling approach, we observed that CsGT45 and MtUGT71G1 share higher structural similarity, indicative of similar binding modes with sugar donors. In addition, the residues that interact with the UDP-Glc molecule were conserved in CsGT45. To our knowledge, no GTase has been shown to use UDP-sophoroside as a sugar donor. Furthermore, the bright blue or red flowers in the Japanese morning glory (Ipomoea nil) contain anthocyanidin 3-O-sophoroside derivatives, and the UDP-glucose:anthocyanidin 3-O-glucoside-2"-O-glucosyltransferase (3GGT) enzyme mediates the glucosylation of anthocyanidin 3-O-glucosides to yield anthocyanidin 3-O-sophorosides, whereas another glucosyltransferase catalyzes the addition of a glucose molecule to the 3 position [61]. Thus, most probably CsGT45 is responsible for the production of Kaempferol 7-O-glucoside and another GGT could be responsible for the generation of kaempferol 7-O-sophoroside.

The absence of CsGT45 activity toward the C-3 or the C-4' positions of kaempferol indicates that other glucosyltransferases are implicated in the flavonoid glucosylation in *C. sativus *stigma. In fact, enzymes that catalyzed the transfer of glycosyl groups to the flavonol C-3 position are included in a different cluster than CsGT45.

The increase of flavonoids in the stigma tissue could be associated with the role of flavonoids in protection against abiotic and biotic stresses [62]. The ultra-violet (UV)-absorbing characteristics of flavonoids have long been considered to be evidence for the role of flavonoids in UV protection. The mechanism of protection by flavonoids could be suppression of free radicals formed upon exposure to UV light [63]. Flavonoids are often present in the epidermal cell layers of leaves and in tissues that are susceptible to UV light, such as pollen and the apical meristem [64]. In *C. sativus *the flavonoid levels are specially high in scarlet stigmas (-2da to +3da) that are characterized for being exposed to sunlight, whereas the stigmas for earlier developmental stages are under the soil and enclosed inside the perianth tubes, and therefore protected from the light.

Another well-documented property of flavonoids is their antimicrobial effect [65,66]. High levels of kaempferol are even reported to inhibit the growth of viruses [67]. The mechanism of kaempferol toxicity is not known, but kaempferol can promote radical formation that might interfere with vital functions of pathogens [68]. The stigma appears to offer a hostile environment to bacteria and fungi since growth of these organisms on the stigma is rare [69]. The presence of flavonoids in the stigma tissue could be associated with the protection of stigmas from pathogen attack.

In addition to these possible functions of flavonoids in the stigma of *C. sativus*, flavonoids are also involved in the control of polar auxin transport [5,70,71], which determines plant organ morphology, such as leaf and flower shape [72]. In flowers, stigma and style growth is due to cell elongation, but not to cell division. The transition from the red stage of stigma development in *C. sativus *to the fully developed stigma (-2da to +3da) is accompanied by a rapid increase in size. When the stigmas of *C. sativus *are fully developed they are slender at the base and wider at the apex where they fold to give a trumpet-like structure [73]. The typical morphology of *C. sativus *stigmas could be the result of different auxin distribution controlled by flavonoid signals.

Expression analysis showed low levels of *CsGT45 *in tepals. The tepals of *C. sativus *are characterized by high levels of anthocyanins, up to 90% of total flavonoids [17]. Nevertheless, a total of nine quercetin and kaempferol glycosides have been identified in minor amounts in *C. sativus *tepals, and a flavonoid-7-*O*-glucosyltransferase was predicted to be responsible for the formation of two of them [17].

## Conclusion

In this study, we have determined the role of CsGT45 in the transfer of glucose on 7-OH of flavonoids, together with its implication in the generation of *C. sativus *flavonoids in the stigma tissue. *C. sativus *stigmas are mainly used for culinary purposes, and historically have been employed in many medicinal drugs against numerous health conditions [74,75]. Flavonoids have long been known to be important nutraceutical components in our diet, due to their potent anti-oxidant properties [76], with glycosylation as a major mechanism influencing their activity. Therefore, the characterization of glucosyltransferases implicated in the formation of these compounds in *C. sativus *will help to understand the biosynthesis and regulation of these glucosides and their implications in the nutraceutical properties of saffron.

## Methods

### Chemicals and Plant materials

Chemicals and reagents were obtained from Sigma-Aldrich unless otherwise stated. Plant tissues and stigmas from *C. sativus *grown under field conditions in Tarazona de La Mancha, Spain, were used throughout the experiments. *C. cancellatus*, *C*. *speciosus*, *C. niveus *and *C*. *cartwrightianus *were obtained from Dr. U. Jacobsen from the Agricultural University of Denmark. Stigmas were collected at the developmental stages previously described [77], and defined as follows: yellow stigma, closed bud inside the perianth tubes (around 0.3 cm in length); orange stigma, closed bud inside the perianth tubes (around 0.4 cm in length); red stigma, closed bud inside the perianth tubes (0.8 cm in length); -2da, two days before anthesis, dark red stigma in closed bud outside the perianth tubes (3 cm in length); da, day of anthesis, dark red stigma (3 cm in length); +1da, one day after anthesis, dark red stigma and +3da, three days after anthesis, dark red stigma. Tepals, style and stamens were collected from flowers at the time of anthesis and together with corms were frozen in liquid nitrogen and stored at -80°C until required. To determine stress-induced gene expression in leaves, whole leaves were collected from plants growing in fields, cut into 1 cm-long pieces and transferred to 24-well-plates containing 1 ml water supplemented with abscisic acid (ABA) (100 μM), 2,4-dichlorofenoxiacetic acid (2,4D) (100 μM), Giberellic acid (GA_3_) (100 μM), 0.2 μl/ml Methyl Jasmonate (MetJa), 200 mM CaCl_2_, 1 mM potassium salicylate (SA), pH 6.5, and distilled water that was used as a control. All the samples were incubated under normal conditions (16 h light/8 h dark cycles at 22°C). Wounding was performed on leaves with a sterile needle and samples were then frozen immediately in liquid nitrogen and were stored at -80°C until used.

### Cloning of *C. sativus *GTase cDNA

As a first step in identifying GTases genes expressed in saffron stigmas, total RNA and mRNA were isolated from developed saffron stigmas by using Ambion PolyAtrack and following manufacturer's protocols (Ambion Inc., Austin, TX, USA). First-strand cDNAs were synthesized by reverse transcription (RT) from 2 μg of total RNA using an 18-base pair oligo dT primer and a first-strand cDNA synthesis kit (Amersham Biosciences) according to manufacturer's instructions. These cDNAs were used as templates for PCR using degenerate primers designed based on the conserved regions of the plants GTases [78]. The primers used were: glut-f (5'-TSNGTNGCNTAYGTNTSNTTYGG-3') and glut-r (5'-TTCCANCCRCARTGNGTNACRAA-3'). Anchored PCR with gene-specific primers were used to analyse and identify the 5' and 3' ends of the glucosyltransferases. For these, 1 μg of poly(A)^+ ^RNA from stigmas was used to synthesize the 5' and 3' ends of the first-strand cDNA using Superscript II reverse transcriptase, using the primers 5'-CDS primer and SMARTII-A oligo for the 5'-RACE reaction and the 3'-CDS primer A for the 3'-RACE reaction supplied in the SMART™ RACE cDNA Amplification kit (Clontech-Takara). Following dilution, the first-strand reaction product was subjected to PCR for amplification. We used the gene-specific primers CsGT45-f1 (5'-AGCTGTCGATAAGATGGATATC-3'), and CsGT45-f2 (5'-GAGTGTCTTATCGCATCCT-3') as forward primers, and CsGT45-r1 (5'-GAAGCCAAGCTCTCCATCGTCGA-3') and CsGT45-r2 (5'-CTCCAGCTGGCCGAATGTGTTC-3') as reverse primers in combination with the universal primer mix from the SMART RACE kit as the reverse/forward primer with the following cycling program: one cycle at 94°C for 3 min, 10 cycles at 94°C for 20 s, 66°C–0.2°C/cycle for 20 s, and 72°C for 2 min, 30 cycles at 94°C for 20 s, 64°C for 20 s and 72°C for 2 min, and a final extension at 72°C for 5 min. The amplified PCR products were analysed by electrophoresis in 1% agarose gel. The PCR products were then cloned into pGEM-T (Promega Corporation, Madison, WI, USA). The ligated DNA was transformed into *E. coli *strain JM109. The clones (20 colonies) were picked individually and amplified in 3 ml of LB medium at 37°C overnight. The plasmid DNA from each clone was extracted using a DNA plasmid Miniprep kit (Promega Corporation, Madison, WI, USA) and then analysed by *Eco*RI restriction digestion. One clone of each size was sent to t Macrogen Inc. (Seoul, South Korea) for sequencing using the BigDyeTM terminator kit and run on ABI 3730XL (Perkin Elmer) with either the T7 or Sp6 sequencing primers. Computer-aided sequence similarity searches were made with the BLAST suite of programs at the National Centre for Biotechnology Information (NCBI; ) Motif searches were made using PROSITE , TMPRED , Signal IP  and PSORT II . Once the 5' and 3' sequences were determined, the full-length clone CsGT45 was amplified from the cDNA and genomic DNA with the following primer sequences: the forward primer, 5'-CAGATGGACCAACATCAGCCT-3'; and the reverse primer 5'-ATTATCTCAACACCTGTGTGG-3'.

### Heterologous expression

The full-length open reading frame of CsGT45 cDNA was amplified by PCR using *Pfu *polymerase (Promega Corporation, Madison, WI, USA). The oligonucleotide sequences for CsGT45 cloning were as follows: the forward primer 5'-ACCAACATCAGCCTAACATT-3', and the reverse primer 5'-T**GCGGCCGC**TCCTCCTTTAAGAGGGTGA-3'. Using these primers, the generated product has a *Not*I site at the 3' end (underlined in the reverse primer). The PCR product was cloned directionally (*Sma*I-*Not*I) into bacterial GST expression vector pGEX-5T-3 (Amersham Biosciences/GE Healthcare) to create in-frame fusions at the 5' terminus with the GST coding sequence. The construct was sequenced to confirm that the gene was in the correct reading frame. After transformation into BL21(DE3) *E. coli *cells, colonies were selected on LB containing ampicillin (AMP) plates. Individual colonies were grown overnight in 5 ml of LB-AMP medium at 20°C, and 2.5 ml of the culture was used to inoculate 500 ml of LB-AMP fresh medium. Cells were grown at 20°C until an A_600 _of 0.6 was reached, after which the culture was induced with 0.5 mM IPTG and allowed to grow for 16 h at 20°C. The cells were harvested by centrifugation at 5,000 g for 10 min and resuspended in 20 ml PBS. Resuspended cells were sonicated with a microtip probe in ice until the viscosity disappeared. After sonication, the samples were centrifuged at 10,000 g for 25 min. The supernatant and pellet were tested by PAGE (polyacrylamide gel electrophoresis)/SDS for solubility of the fusion protein by coomasie stain. The soluble proteins were applied to a glutathione Sepharose column for purification following manufacturer instructions (Amersham Biosciences/GE Healthcare). Protein concentration was determined according to the Bradford method [79], using serum albumin as standard.

### Enzyme assays and analysis of reaction products

The affinity-purified enzyme was used to determine substrate specificity and enzymatic parameters. In a final assay volume of 200 μl, the reaction conditions were 50 mM Tris-HCl, pH 7.5, 14 mM 2-mercaptoethanol, 2.5 mM UDP-glucose, the recombinant enzyme (7.0 μg) and the corresponding substrates: 100 μM Quercetin, 100 μM kaempferol, 100 μM cyanidin, 1 mM trans-cinnamic acid, 1 mM sinapic acid, 1 mM indole acetic acid, 1 mM abscisic acid and 1 μM crocetin encapsulated in maltosyl-β-cyclodextrin as described [80]. All the glucosyltranferase activity assays were carried out at 30°C for 30 minutes. For the determination of the *K*_m _values of substrates, the concentrations of kaempferol and quercetin were varied from 20 to 100 μM, at a fixed UDP-glucose concentration of 2.5 mM and 7.0 μg of the purified enzyme. These enzymatic reactions were performed at 30°C for 10, 15 and 20 minutes for each substrate concentration. The reactions were terminated, and the proteins precipitated, by the addition of 20 μl of trichloroacetic acid (240 mg/ml). Subsequently, samples were centrifuged at 15,000 g for 5 min to collect the supernatant, and aliquots were analysed by reverse-phase HPLC as previously described [81] using a C18 Ascentis, 25 × 4.6, particle size 5 um column (Supelco, Sigma-Aldrich). *K*_m _values were determined from Lineweaver-Burk plots of initial rate data. The assays were also analysed by HPLC DAD detector and by electrospray ionization (ESI)-mass spectrometry (MS) for the formation of glycosylated products as previously described [81] using a C18 Ascentis, 25 × 4.6, particle size 3 um column (Supelco, Sigma-Aldrich). A standard curve for peak area of quercetin and kaempferol was produced by injecting known amounts of these flavonoids. For individual quercetin glucosides, standard curves were constructed indirectly by calculating the amount of quercetin or kaempferol released after quantitative hydrolysis of the glucoside with β-glucosidase from almonds (60 min, pH 6, 30°C). The assignment of a glucosylation position to quercetin and kaempferol was determined indirectly following the method by Mabry *et al*. [50] for the identification of flavonoids, using published data [31,47,51] and by comparison with authentic flavonol standards: kaempferol 7-*O*-glucoside and quercetin 4'-*O*-glucoside (TransMIT Flavonoidforschung, DE) and quercetin 3-*O*-glucoside (Sigma-Aldrich).

### Flavonoid analysis in stigma tissue

Stigmas at the time of anthesis were ground in liquid nitrogen. The fine powder obtained was extracted with methanol (500 μl), centrifuged and the supernatant analysed by LC-ESI-MS using a C18 Ascentis column 15 × 2.1, particle size 3 um (Supelco, Sigma-Aldrich) and following the method previously described [81]. For flavonoid analysis from stigmas at different developmental stages (yellow to +3da), three stigmas of each stage were collected and freeze-dried. The powder obtained from one stigma was extracted with 500 μl of methanol containing 0.2 mg/ml 4-methylumbelliferyl β-D-glucuronide as an internal standard. The samples were centrifuged (5,000 g, 10 min), and the supernatant evaporated and treated as described [15]. Samples in triplicate were analysed by HPLC as described [81] using a C18 Ascentis, 25 × 4.6, particle size 5 um column (Supelco, Sigma-Aldrich).

The nuclear magnetic resonance (NMR) spectra were recorded on Brucker DRX 500 NMR instrument operating at 500 MHz for ^1^H and at 125 MHz for ^13^C, respectively. Chemical shifts were recorded as described [15].

### Analysis of mRNA levels in different tissues and stress conditions

Reverse Transcription-PCR (RT-PCR) was used to determine the relative levels of *CsGT45 *and *UGTCs3 *messages. Total RNA was isolated from control and treated leaves, tepals, stamens, stigmas, styles and corms using the Trizol reagent (Gibco-BRL). The RNA was resuspended in 100 μl of RNase-free water and treated with RQ1 RNase-free DNase (Promega Corporation, Madison, WI, USA). The DNase was heat inactivated before RT-PCR. The RNA was quantified with a spectrophotometer and stored at -80°C. Various initial concentrations of mRNA, ranging over 10 fold difference, were used to demonstrate the differential accumulation of the mRNA in the tissues analysed. First-strand cDNAs were synthesized by RT from 2 μg of total RNA using a first-strand cDNA synthesis kit (Pharmacia) and random primers. Conditions for semi-quantitative RT-PCR were as follows: 65°C for 5 min, followed by 37°C for 1 h, followed by 75°C for 5 min. The cDNAs obtained were used as templates for PCR using the *CsGT45 *gene-specific primers: 5'-GATGGGGAGAGAGGTGTTGA-3' and 5'-TCCTCGCAATGCTGTCTATG-3', and for the amplification of gene coding for the 18S ribosomal RNA (RPS18) the primers used were: 5'-AGTTTGAGGCAATAACAGGTCT-3' and 5'-GATGAAATTTCCCAAGATTACC-3'. *UGTCs3 *was amplified using the primers previously described [52]. Thermal cycling parameters were 2 min at 95°C, 30 × (20 s at 95°C, 20 s at 60°C, and 30 s at 72°C). The PCR products were separated in a 2% agarose gel. The gels were photographed with the IP-010-Sd photo-documentation system (Vilber Lourmat). The PhotoCaptMw programme was used to quantify the intensity of the ethidium bromide stained DNA bands from the positive images of the gel. These experiments were repeated three times and averaged for each sample. To correct the initial mRNA levels, each intensity score was normalized to the intensity for the RPS18 gene amplification.

## List of abbreviations

C4H: cinnamate-4-hysroxylase; GTases: glycosyltransferases; HPLC-ESI-MS: HPLC-electrospray ionization-mass spectrometry; PAL: L-phenylalanine ammonia lyase;. RACE: rapid amplification of cDNA ends; RT-PCR: reverse transcription-PCR; UDP: uridine diphosphate glucose; UGT: UDP-glycosyltransferase.

## Authors' contributions

ARM carried out the HPLC analyses; LGG contributed to design and carry out the experiments and wrote the paper; ATM did the cloning of CsGT45 ORF in the expression vector and made contribution to the expression analysis; OA participated in the cloning of CsGT45, in the RT-PCR experiments and review the manuscript; LGG conceived of the study, and participated in its design and coordination, helped in the RT-PCR experiments, performed the activity assays and draft the manuscript. All authors have read and approved the final manuscript
